# Efficacy of serratus anterior plane block on postoperative nausea and vomiting: a meta-analysis of randomized controlled trial

**DOI:** 10.3389/fmed.2026.1867333

**Published:** 2026-08-12

**Authors:** Jinfang Zeng, Zijian Li, Han Gao, Ying Ye, Jieyu Hua, Dongxiao Huang

**Affiliations:** 1 Department of Anaesthesiology and Pain Medicine, Jiangnan University Medical Center, Wuxi No. 2 People’s Hospital, China; 2 Jiangsu Province Key Laboratory of Anesthesiology and Jiangsu Province Key Laboratory of Anesthesia and Analgesia Application Technology, NMPA Key Laboratory for Research and Evaluation of Narcotic and Psychotropic Drugs, Xuzhou Medical University, Xuzhou, Jiangsu, China; 3 Wuxi School of Medicine, Jiangnan University, Wuxi, China

**Keywords:** meta-analysis, PONV, postoperative nausea and vomiting, randomized controlled trial, SAPB, serratus anterior plane block

## Abstract

**Background:**

Postoperative nausea and vomiting (PONV) still often occurs after surgery, which adversely affects recovery and patient satisfaction. Serratus anterior plane block (SAPB) has been proposed as a regional anesthesia technique that may reduce PONV indirectly by improving postoperative analgesia and decreasing perioperative opioid requirements.

**Methods:**

A systematic review and meta-analysis of the randomized controlled trial (RCT) was conducted to evaluate the impact of SAPB on PONV. From its establishment to April 20, 2026, the central register of PubMed, Embase, CNKI and Cochrane controlled trials was searched. Eligible studies include adults who underwent surgery under general anesthesia with a control group (placebo or no intervention). The main results are the incidence of postoperative nausea and vomiting; the secondary results include postoperative opioid consumption, pain score (visual analog scale, VAS), hospitalization time, the time of first rescue analgesia, and the incidence of headache or hypotension. Random effect element analysis was carried out to gather risk differences (RD) and standardized mean differences (SMD) with 95% confidence intervals (CI). Use GRADE to evaluate the certainty of evidence and apply test sequence analysis (TSA) to evaluate robustness.

**Results:**

Twenty RCTs involving 1,651 patients were included. Compared with control treatment, SAPB was associated with a reduced incidence of postoperative nausea (RD = −0.17; 95% CI: −0.23 to −0.11; *I*^2^ = 67%; moderate-certainty evidence). SAPB may also reduce postoperative vomiting, although this finding was borderline and should be interpreted cautiously (RD = −0.13; 95% CI: −0.25 to −0.00; *I*^2^ = 79%; moderate-certainty evidence). SAPB reduced fentanyl consumption (SMD = −1.08; 95% CI: −1.86 to −0.31) and remifentanil consumption (SMD = −2.14; 95% CI: −3.25 to −1.03), whereas sufentanil consumption was not significantly reduced. SAPB also improved postoperative pain scores, shortened hospital stay, prolonged the time to first rescue analgesia, and reduced headache incidence. Funnel plot inspection and Egger’s and Begg’s tests did not suggest significant small-study effects for postoperative nausea. Risk-of-bias concerns were mainly related to insufficient reporting of allocation concealment and blinding procedures.

**Conclusion:**

SAPB appears to reduce postoperative opioid requirements and improve analgesia in patients undergoing breast and thoracic surgery. These benefits are associated with a reduced incidence of postoperative nausea and may contribute to enhanced recovery. However, the current evidence does not establish an independent direct antiemetic effect of SAPB, and the effect on postoperative vomiting should be interpreted cautiously. Further high-quality RCTs with standardized opioid and antiemetic protocols are needed to clarify the causal pathway between SAPB, opioid reduction, and PONV.

**Systematic review registration:**

https://www.crd.york.ac.uk/prospero/CRD420261373467, identifier CRD420261373467.

## 1 Introduction

In recent years, the idea of Enhanced Recovery After Surgery has been increasingly advocated and put into practice across healthcare settings, and has been increasingly accepted by both patients and medical staff. Postoperative nausea and vomiting (PONV) is still a common and clinically significant problem among surgical patients. The reported incidence rate ranges from about 30% to as high as 80%, depending on the individual risk profile (1, 2). In addition to causing considerable discomfort, PONV is also associated with a series of adverse consequences, including lung inhalation, fluid consumption, electrolyte disorders and prolonged postoperative recovery time. These complications will eventually lead to prolonged hospitalization and increased health care spending (3, 4). Although drug prevention—especially the combination of ondansetron and dexamethasone—is widely used in clinical practice, its effectiveness is not absolute. In addition, concerns about potential adverse effects highlight the need to explore additional or alternative prevention methods (5).

Serratus anterior plane block (SAPB) has emerged as a promising regional anesthesia technique that may contribute to lower PONV risk by improving postoperative analgesia and reducing perioperative opioid exposure. In contemporary clinical anesthesia practice, ultrasound-guided SAPB has become the preferred and mainstream method, because it improves safety, precise needle placement and consistent local anesthesia diffusion, and minimizes the risk of complications such as pneumothorax and neurovascular damage (6). SAPB is a mature fascia plane blocker, which exerts a targeted analgesic effect by depositing local anesthetics in the potential space between the serrated anterior muscle and the underlying ribs, thus effectively blocking the lateral skin branch of the intercostal nerve, thoracic dorsal nerve and long thoracic nerve that dominate the anterior chest wall (6, 7). As the core component of the multi-mode analgesia program, SAPB is mainly used for perioperative and postoperative analgesia of thoracic surgery, breast surgery and other chest wall-related surgeries, providing continuous pain relief and minimizing systemic side effects. Several clinical studies (8, 9) confirmed that SAPB significantly reduced the postoperative pain score during rest and activity, as well as the total consumption of opioids in the perioperative period, which contributed to smoother postoperative recovery (10). Accordingly, several randomized controlled trials recorded that SAPB effectively reduced the need for opioids such as morphine and suphentanyl in patients undergoing chest and breast surgery, and optimized the overall analgesic characteristics (11).

This meta-analysis attempts to fill this gap by integrating the results of 20 randomized controlled trials and aims to evaluate how SAPB affects the occurrence of PONV. Further group analysis was carried out to explore the potential impact of variables such as the type and dosage of local anesthetics, as well as the differences in surgical procedures. The purpose of this system review and meta-analysis is to determine the impact of SAPB on PONV risk. The findings aim to guide the evidence-based use of SAPB in a wide range of multi-model methods to reduce PONV.

## 2 Materials and methods

In accordance with the PRISMA statement, we performed a thorough meta-analysis to examine the impact of SAPB on PONV. The protocol was registered in PROSPERO (CRD420261373467). Since this research compiled and analyzed prior studies, neither ethics committee approval nor patient consent was needed. This study focused on the occurrence of postoperative nausea (PON) and vomiting (POV) as the primary outcome. PON was characterized by nausea alone or with vomiting, whereas POV was characterized by vomiting alone or in combination with nausea. When PONV was reported as a composite outcome, it was analyzed as postoperative nausea because this category included patients with nausea, with or without vomiting. On the contrary, studies that only recorded PON (i.e., nausea without vomiting) did not serve as an alternative to PONV results to avoid incorrectly including cases involving vomiting. In order to evaluate the effectiveness of antiemetic intervention, the results are standardized to 24 h after surgery. For research that reports different observation windows, choose the time point closest to 24 h for analysis.

### 2.1 Search approach and eligibility standards

A systematic literature search was conducted in PubMed, Embase, the Cochrane Central Register of Controlled Trials, and China National Knowledge Infrastructure (CNKI) from database inception to April 20, 2026. The search strategy combined intervention-related terms (“serratus anterior plane block,” “serratus plane block,” or “SAPB”), outcome-related terms (“postoperative nausea and vomiting,” “PONV,” “nausea,” or “vomiting”), and study design-related terms (“randomized controlled trial,” “RCT,” or “random allocation”). Boolean operators were applied, and database-specific indexing terms, such as MeSH terms in PubMed, were used when appropriate. No language restriction was applied in order to minimize language bias and improve the comprehensiveness of the evidence base. Records published in languages other than English were screened using the same eligibility criteria. One Chinese-language randomized controlled trial identified through CNKI was reviewed by investigators fluent in both Chinese and English, and data were extracted using the same standardized extraction form as that used for English-language studies. Reference lists of relevant articles and reviews were also manually checked. The detailed search strategy for each database is provided in Supplementary Table 1.

### 2.2 Research selection

The study extracted data on the authors’ identities, publication year, anesthesia and surgical characteristics (type and duration), intervention details, occurrences of nausea or vomiting, and total participant numbers. Two authors (H.J.Y and Y.Y.) independently screened the articles for eligibility, with any discrepancies resolved through discussion among all authors.

### 2.3 Inclusion criteria

Studies were included if they satisfied all predefined criteria: (1) population—adult patients undergoing surgical procedures under general anesthesia; (2) intervention—administration of SAPB using local anesthetics; (3) comparator—placebo, sham block, saline injection, no regional block, or no intervention; (4) outcomes—postoperative nausea and/or vomiting as the primary outcomes, with secondary outcomes including visual analog scale scores, postoperative opioid consumption, length of hospital stay, time to first rescue analgesia, and adverse events; and (5) study design—randomized controlled trials.

### 2.4 Exclusion criteria

Studies were excluded if they met any of the following criteria: (1) available only as trial registration records, conference abstracts, case reports, reviews, or non-original articles; (2) insufficient data for quantitative synthesis; (3) inappropriate or flawed statistical reporting; (4) absence of a placebo, sham, saline, no-block, or no-intervention control group; (5) comparison of SAPB only with another active regional anesthetic technique; or (6) enrollment of pediatric or adolescent patients unless adult data could be extracted separately.

### 2.5 Information extraction and assessment of bias risk

Using the Cochrane Collaboration’s Risk of Bias tool, version 1 (RoB 1), two investigators (L.Z.J. and G.H.) independently assessed the methodological quality of the included studies. The assessed domains included random sequence generation, allocation concealment, blinding of participants and personnel, blinding of outcome assessment, incomplete outcome data, selective outcome reporting, and other potential sources of bias. Each domain was judged as having a low, unclear, or high risk of bias. Disagreements were resolved through discussion with a third investigator. To examine the influence of methodological quality on the primary outcome, sensitivity analyses were performed by excluding studies judged to be at overall high risk of bias. The pooled estimates after excluding high-risk studies were compared with the main analysis.

### 2.6 Evidence quality assessment

Using the GRADE system and the Guideline Development Tool, the overall reliability of the evidence was systematically evaluated. Following GRADE guidance, an assessment was performed to determine the likelihood of systematic and random errors and the overall reliability of the evidence. Evidence quality was assessed and assigned to one of four categories—extremely low, low, moderate, or high—considering bias, result variability, imprecision, publication bias, and the magnitude of treatment effects.

### 2.7 Trial sequential analysis

TSA was conducted using version 0.9.5.10 beta of the TSA software to assess the robustness of cumulative evidence and the need for additional studies. The diversity-adjusted required information size, accounting for between-study heterogeneity, was calculated with a predefined two-sided type I error of 5% and type II error of 20%. The cumulative Z-curve was compared with sequential monitoring boundaries, and evidence was considered conclusive if it crossed the boundary before reaching the required information size; otherwise, further trials were deemed necessary to confirm the findings.

### 2.8 Outcome measures

A *Z*-test was used to evaluate the overall effect, with statistical significance defined as *p* < 0.05. For all analyses, a random-effects model was utilized. The efficacy of SAPB in reducing postoperative nausea and vomiting was assessed via the pooled RD, taking into account administration timing, surgery type, and local anesthetic details, including type, concentration, and dosage.

For dichotomous outcomes, including postoperative nausea, vomiting, headache, hypotension, and pruritus, pooled risk differences or risk ratios with 95% confidence intervals were calculated. For continuous outcomes, including visual analog scale pain scores, opioid consumption, length of hospital stay, and time to first rescue analgesia, pooled mean differences or standardized mean differences with 95% confidence intervals were calculated as appropriate. Exploratory subgroup analyses were performed to investigate potential sources of clinical heterogeneity. Subgroups were analyzed according to surgical category (breast surgery vs. thoracic surgery), SAPB plane when clearly reported (superficial SAPB vs. deep SAPB), local anesthetic type, concentration, dose, and timing of SAPB administration. Subgroup analyses according to surgical approach (open vs. minimally invasive procedures) and SAPB administration mode (single-shot vs. continuous SAPB) were planned but were not pooled because these variables were not consistently or explicitly reported across the included trials. We avoided assigning studies to these subgroups by inference alone in order to reduce misclassification bias. To assess the robustness of the results, leave-one-out sensitivity analyses were performed on studies with low or unclear risk of bias, and statistical heterogeneity was evaluated using the I^2^ statistic.

Publication bias and small-study effects were assessed for outcomes including at least 10 studies. Funnel plots were visually inspected for asymmetry, and Egger’s regression test and Begg’s rank correlation test were performed when applicable. For outcomes with fewer than 10 studies, formal tests for publication bias were not performed because of insufficient statistical power and the risk of misleading results.

For multi-arm trials with more than one SAPB intervention group sharing the same control group, intervention groups were combined according to Cochrane recommendations before meta-analysis to avoid double-counting of the shared control group.

Clinical relevance was interpreted descriptively rather than used as a formal criterion for statistical significance. For continuous analgesic outcomes, the magnitude of effect was considered in relation to commonly used clinically meaningful thresholds, including an approximately 1-point reduction in visual analog scale pain scores and a relative reduction of approximately 30% in opioid consumption. These thresholds were used only to contextualize the clinical importance of statistically significant findings and were not used to determine study eligibility or the primary meta-analytic conclusions.

Opioid consumption was extracted according to the opioid agent, route of administration, dose unit, and postoperative time window reported in each trial. When the reported data were sufficiently comparable, postoperative opioid consumption was converted to intravenous morphine-equivalent doses and pooled as a standardized opioid-sparing outcome. When conversion was not clinically appropriate because of substantial differences in opioid pharmacokinetics, administration phase, reporting units, or time windows, opioid consumption was analyzed separately according to opioid type. In particular, remifentanil consumption was retained as a separate intraoperative opioid-sparing outcome because it is usually administered as a short-acting intraoperative infusion and is not directly comparable with postoperative morphine-equivalent analgesic consumption.

## 3 Results

### 3.1 Study selection

**FIGURE 1:**
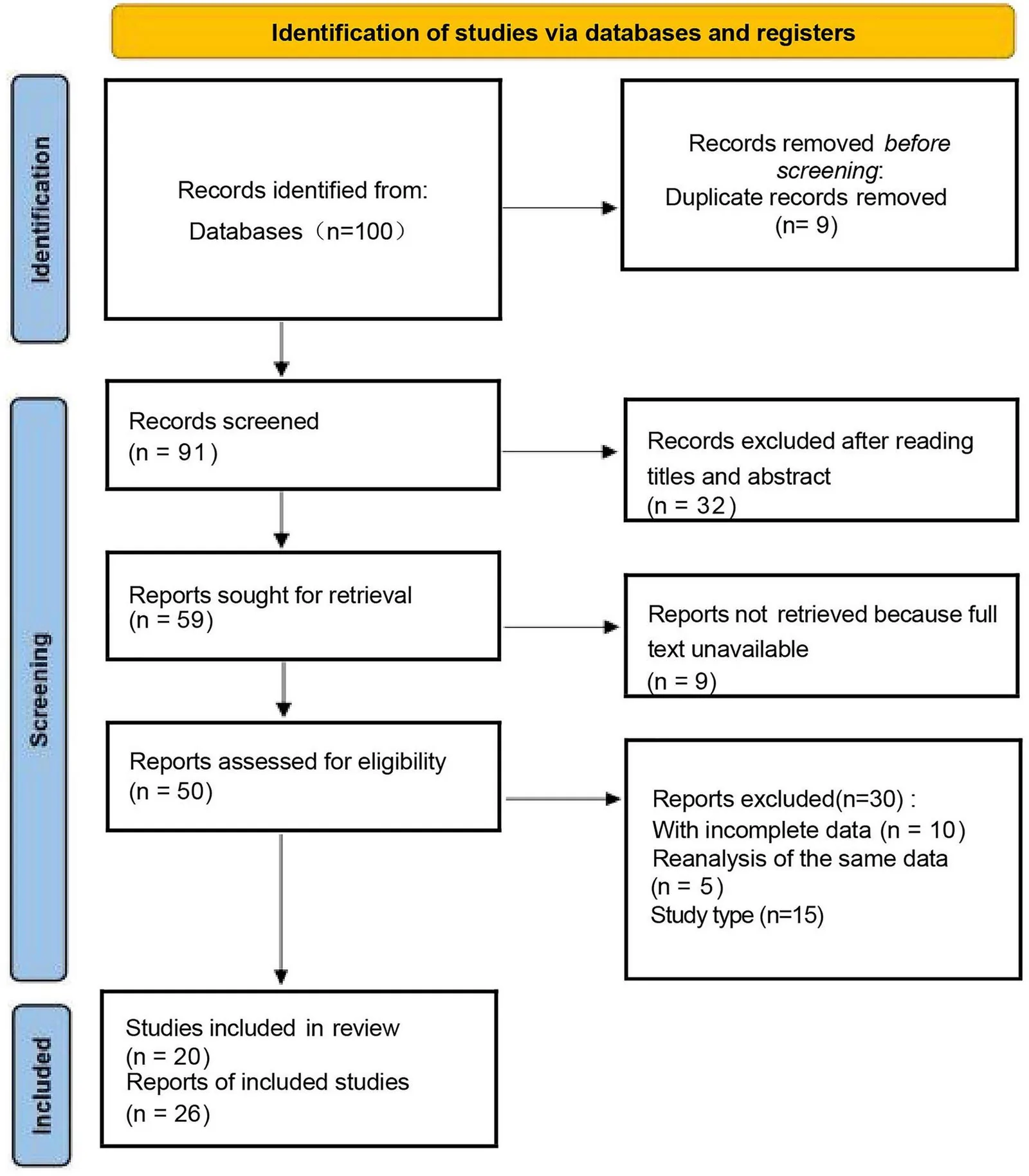
PRISMA flow diagram of study selection. The numbers of records identified, screened, assessed for eligibility, excluded, and included in the final meta-analysis are shown.

Figure 1 shows the study selection process. A total of 100 records were identified from database searches (PubMed, Embase, Cochrane Library, CNKI). After removing 9 duplicate records, 91 records remained for screening. Based on the review of titles and abstracts, 32 records were excluded, leaving 59 reports sought for retrieval. Among these, 9 reports were not retrieved because the full text was unavailable. Therefore, 50 reports were assessed for eligibility. During the full-text assessment, 30 reports were excluded, including 10 with incomplete data, 5 involving reanalysis of the same data, and 15 with an ineligible study type. Finally, 20 studies (12–31), corresponding to 26 reports, were included in the review.

### 3.2 Study characteristics

Across the included literature, 20 randomized controlled studies (12–31) evaluated the efficacy of SAPB in preventing (PONV, involving a total of 1,651 patients (Table 1). The included studies were published between 2017 and 2026. Regarding the local anesthetic used for SAPB, bupivacaine was used in six studies, involving 229 patients in the SAPB groups, whereas ropivacaine was used in fourteen studies, involving 676 patients in the SAPB groups. Most procedures were related to breast and thoracic surgery, including 775 patients undergoing breast surgery and 817 patients undergoing thoracic surgery; one study involving 59 patients focused on minimally invasive cardiac surgery. In terms of timing, SAPB was most commonly administered before surgery or after induction of anesthesia, although several studies performed the block after completion of surgery or in the recovery room.

**TABLE 1:** General information of patients with incidence of postoperative nausea and vomiting.

References	Age	Sex (M/F)	Type of surgery	Surgical category	Anesthetic type	SAPB level	Sensory testing/block verification	Comparisons (Group)	Time of administration	Nausea	Vomiting	Total
Bakeer et al. (12)	18–60 years	0/58	Modified radical mastectomy	Breast	General anesthesia (sevoflurane inhalation + remifentanil)	Unilateral (T2–T6)	Pinprick test performed	SAPB group 0.25% bupivacaine 30 mL	Before general anesthesia induction	4	−	58
		0/58						Control group 0.9% saline 30 mL		35	−	58
Akgün et al. (13)	18–65 years	0/32	Modified radical mastectomy	Breast	General anesthesia (propofol+ fentanyl+ sevoflurane)	Unilateral (T3–T9)	Not mentioned	SAPB group 0.25% bupivacaine 20 mL	Preoperative	7	−	32
		0/32						Control group 0.9% saline 20 mL		8	−	32
Antonio et al. (14)	> 18 years	12/14	Right minithoracotomy	Minimally Invasive Cardiac	General anesthesia (propofol+ fentanyl+ sufentanil+ remifentanil)	Unilateral (T5)	Not mentioned	SAPB group 0.375% ropivacaine 150 mg	Preoperative	3	−	33
		16/17						Control Group 0.9% saline 30 mL		6	−	26
Qian et al. (15)	18–65 years	0/90	Modified radical mastectomy	Breast	General anesthesia (sufentanil+ propofol+ sevoflurane)	Unilateral (T4–T5)	Not mentioned	SAPB group 0.5% ropivacaine 30 mL	Before general anesthesia induction	3	−	90
		0/89						Control Group 0.9% saline 30 mL		11	−	89
Mensah et al. (16)	18–70 years	0/25	Simple mastectomy	Breast	General anesthesia(fentanyl +propofol+N2O)	Unilateral(T5)	Not mentioned	SAPB Group 0.25% bupivacaine 0.4 mL/kg	After induction of anesthesia	1	−	25
		0/25						Control group 0.9% saline 0.4 mL/kg		2	−	25
Shokri and Kasem (23)	40–56 years	0/23	Simple mastectomy	Breast	General anesthesia (fentanyl+ isoflurane)	Unilateral (T5–T6)	Not mentioned	SAPB group 0.4 mL/kg 0.25% bupivacaine	After completion of surgery	0	−	23
		0/23						Control group 0.9% saline 0.4 mL/kg		4	−	23
Jackson et al. (17)	> 18 years	22/24	VATS/RATS anatomic lung resection	Thoracic	General anesthesia(sevoflurane +fentanyl)	Unilateral(T5–T8)	Not mentioned	SAPB group 0.25% bupivacaine 100 mg	After completion of surgery	6	−	46
		14/32						Control Group 0.9% saline 40 mL		7	−	46
Lee et al. (18)	18–80 years	12/11	Thoracoscopic lobectomy	Thoracic	General anesthesia (propofol+ remifentanil)	Unilateral (T5)	Not mentioned	SAPB group 0.375 % ropivacaine 20 mL	Preoperative	2	−	23
		16/7						Control group 0.9% saline 20 mL		2	−	23
Qiu et al. (19)	32–78 years	11/10	Thoracoscopic lobectomy	Thoracic	General anesthesia (propofol+ sufentanil+ remifentanil)	Unilateral (T4–T5)	Not mentioned	sSAPB group 0.375% ropivacaine 0.4 mL/kg	After completion of surgery	5	−	21
		9/12						dSAPB group 0.375% ropivacaine 0.4 mL/kg		4		21
		13/8						Control group 0.9% saline 0.4 mL/kg		7	−	21
Li et al. (20)	40–70 years	12/14	Thoracoscopic lobectomy	Thoracic	General anesthesia(propofol +sufentanil)	Unilateral(T3–T9)	Not mentioned	SAPB Group 0.375% ropivacaine 40 mL	In the recovery room	6	−	26
		13/14						Control Group 0.9% saline 40 mL		10	−	27
Park et al. (21)	20–80 years	18/24	Thoracoscopic pulmonary resection	Thoracic	General anesthesia (sevoflurane+ remifentanil)	Unilateral (T5–T7)	Loss of cold sensation test	SAPB Group ropivacaine 0.375% 30 mL	After induction of anesthesia	13	1	42
		17/25						Control group 0.9% saline 30 mL		19	1	42
Reyad et al. (22)	> 18 years	31/14	Thoracoscopic lobectomy	Thoracic	General anesthesia (propofol+ fentanyl+ sevoflurane)	Unilateral (T2–T9)	Pinprick test performed	SAPB group 0.25% bupivacaine 20 mL	After completion of surgery	0	−	45
		29/15						Control group 0.9% saline 20 mL		9	−	44
Wang et al. (24)	> 18 years	0/50	Radical mastectomy	Breast	General anesthesia (TIVA propofol+ sufentanil+ remifentanil)	Unilateral (T2–T6)	Ice test performed	SAPB group 0.375% ropivacaine 20 mL	Before induction of anesthesia	11	2	50
		0/50						Control group 0.9% saline 20 mL		25	7	50
Wang et al. (25)	32–56 years	0/26	Radical mastectomy	Breast	General anesthesia (propofol+ sufentanil+ sevoflurane)	Unilateral (T4–T5)	Not mentioned	SAPB group 0.5% ropivacaine 20 mL	After induction of anesthesia	4	–	26
		0/26						Control group 0.9% saline 20 mL		15	–	26
Wanlun et al. (26)	18–75 years	0/22	Radical mastectomy	Breast	General anesthesia (sufentanil+ sevoflurane)	Unilateral (T4–T5)	Not mentioned	dSAPB group 0.5% ropivacaine 20 mL	After completion of surgery	3	–	22
		0/22						Control group 0.9% saline 20 mL		9	–	22
		0/22						sSAPB group 0.5% ropivacaine 20 mL		3	–	22
Chen et al. (27)	20–75 years	17/21	Uniportal VATS pulmonary lobectomy	Thoracic	General anesthesia (propofol+ remifentanil+ sufentanil)	Unilateral (T4–T5)	20 min post-injection, sensory block of skin dermatomes	SAPB group (S) 0.375% ropivacaine 30 mL	Before induction of anesthesia	7	–	38
		22/20						Control group 0.9% saline 30 mL		15	–	42
		12/28						SAPB group (H) 0.375% ropivacaine 30 mL + hydromorphone 1 mg		5	–	40
Zhang et al. (28)	18–85 years	25/18	VATS pulmonary lobectomy	Thoracic	General anesthesia (propofol+sevoflurane + remifentanil + sufentanil)	Unilateral (T2–T9)	Not mentioned	SAPB(R) Group 0.5% ropivacaine 25 mL	After completion of surgery	9	7	43
		23/20						Control Group 0.9% saline 25 mL		18	13	43
		26/17						SAPB(RD) group 0.5% ropivacaine + 1 μg/kg dexmedetomidine 25 mL		4	2	43
Zhang et al. (29)	18–65 years	11/15	VATS pulmonary lobectomy	Thoracic	General anesthesia (propofol+ sevoflurane+ remifentanil+ sufentanil)	Unilateral (T4–T5)	Skin cold perception test	SAPB(R) group 0.5% ropivacaine 25 mL	Preoperative	8	–	26
		10/15						Control Group 0.9% saline 25 mL		8	–	25
		11/16						SAPB(RD) group 0.5% ropivacaine 25 mL		2	–	27
Zhang et al. (30)	> 18 years	0/51	Radical mastectomy	Breast	General anesthesia (propofol+ sufentanil+ sevoflurane+ remifentanil)	Unilateral (T5)	Not mentioned	SAPB Group 0.375% ropivacaine 75 mg	After the induction of anesthesia	2	−	51
		0/51						Control group 0.9% saline 20 mL		5	−	51
Zhao et al. (31)	25–79 years	22/10	VATS pulmonary lobectomy	Thoracic	General anesthesia (propofol+remifentanil)	Unilateral (T2–T8)	Pinprick test performed	SAPB Group 0.4% ropivacaine 20 mL	After induction of anesthesia	2	−	32
		23/8						Control group 0.9% saline 20 mL		8	−	31

### 3.3 The methodological quality of the included studies

**FIGURE 2:**
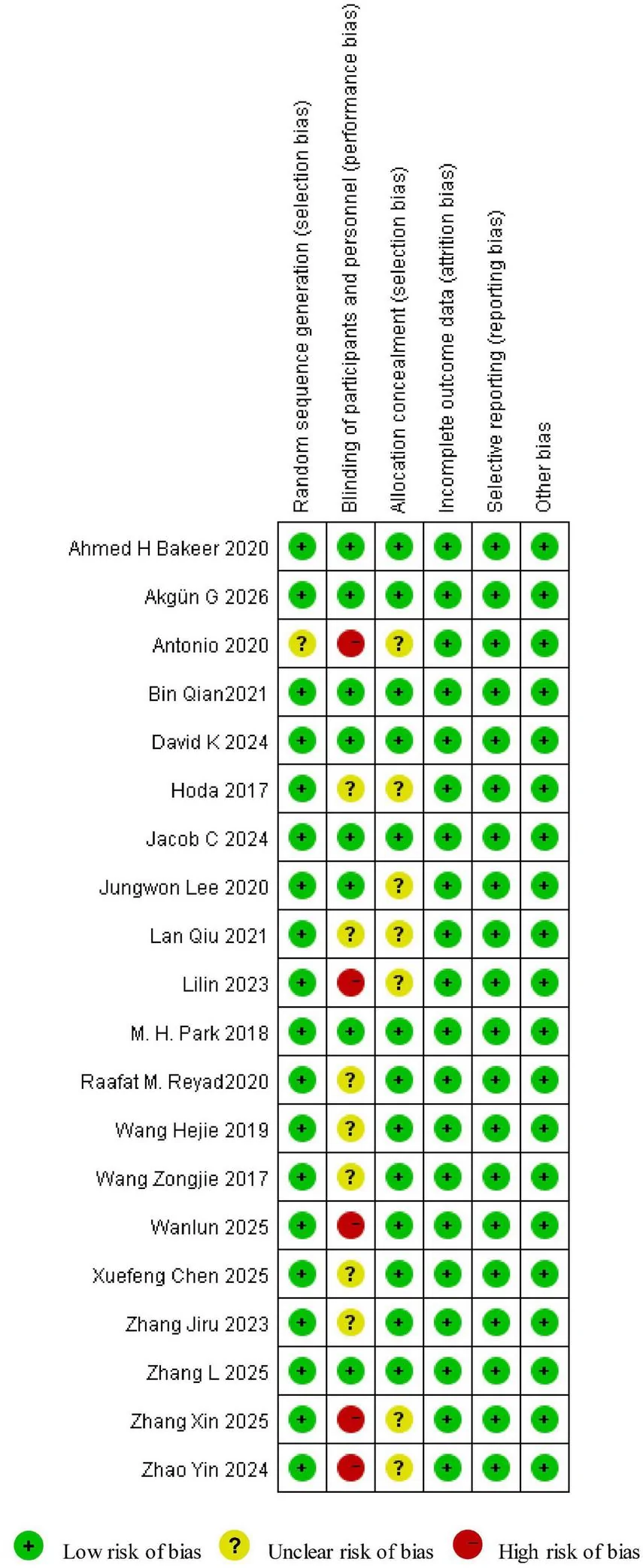
Risk-of-bias summary of the included randomized controlled trials assessed using the Cochrane Risk of Bias tool, version 1 (RoB 1). Each domain was judged as low, unclear, or high risk of bias.

A total of 20 (12–31) randomized controlled trials were assessed using the Cochrane Risk of Bias tool, version 1 (RoB 1). The detailed domain-level judgments are provided in Supplementary Table 2, and the overall risk-of-bias summary is shown in Figure 2. Overall, the included studies showed variable methodological quality. Random sequence generation was adequately reported in several studies, whereas allocation concealment was frequently insufficiently described and was therefore often judged as unclear risk. Blinding of participants, personnel, and outcome assessors was inconsistently reported across trials, which may be particularly important in regional anesthesia studies because inadequate blinding can introduce performance and detection bias. Incomplete outcome data and selective reporting were generally judged as low risk in most studies. The main methodological limitations were insufficient allocation concealment and incomplete reporting of blinding procedures.

### 3.4 Quality of evidence

Evidence certainty was assessed using the GRADE approach. Overall, the certainty of evidence varied across outcomes, mainly because of methodological limitations, including lack of allocation concealment and lack of blinding, as well as statistical heterogeneity and imprecision in several outcomes. For postoperative nausea, 25 comparisons involving 905 patients in the SAPB group and 899 patients in the control group showed that SAPB reduced the risk of nausea, and the certainty of evidence was rated as moderate. The evidence for postoperative vomiting was also rated as moderate. Hospital duration, remifentanil dose, fentanyl consumption, and VAS scores at rest within 0–2 h were rated as low-certainty evidence. Sufentanil consumption was rated as high-certainty evidence. In contrast, hypotension, headache, VAS scores during activity within 0–2 h, itching, and time to first rescue analgesic were rated as very low-certainty evidence (Table 2).

**TABLE 2:** GRADE summary of efficacy of SAPB on PONV.

Outcome	No. of comparisons	Participants (SPAB/control)	Effect estimate (RD/SMD, 95% CI)	Absolute effect	Certainty of evidence (GRADE)	Rationale for downgrading/upgrading
Nausea (PON)	25	116/905 vs. 280/899	RD −0.17 (−0.23 to –0.11)	174 fewer per 1,000 (from 109 fewer to 230 fewer)	Moderate	Downgraded for lack of allocation concealment and lack of blinding, and for moderate/high statistical heterogeneity. Upgraded for strong association (RR < 0.5), which may support increased confidence in the effect estimate when consistent with other domains.
Vomiting (POV)	5	14/210 vs. 42/210	RD −0.13 (−0.25 to 0.00)	104 fewer per 1,000 (from 190 fewer to 21 more)	Moderate	Downgraded for lack of allocation concealment and lack of blinding. No serious inconsistency, indirectness, or imprecision was identified.
Hospital duration (d)	4	234 vs. 230	MD –0.71 lower (−1.07 to−0.34 lower)	−	Low	Downgraded for lack of allocation concealment and lack of blinding, and for imprecision because the total population size was less than 400.
Hypotension	5	20/154 vs. 11/143	RD 0.04 (−0.01 to 0.09)	43 more per 1,000 (from 10 fewer to 90 more)	Very low	Downgraded for lack of allocation concealment and lack of blinding, moderate/high statistical heterogeneity, and imprecision because the total population size was less than 400.
Headache	6	6/255 vs. 31/256	RD −0.09 (−0.18 to −0.00)	94 fewer per 1,000 (from 180 fewer to 0 more)	Very low	Downgraded for lack of allocation concealment and lack of blinding, moderate/high statistical heterogeneity, and imprecision because the total population size was less than 400.
Remifentanil dose	8	283 vs. 279	SMD 2.14 lower (3.25 to 1.03 lower)	−	Low	Downgraded for moderate/high statistical heterogeneity and imprecision because the total population size was less than 400. No serious risk of bias or indirectness was identified.
Fentanyl consumption	4	176 vs. 177	SMD 1.08 lower (1.86–0.31 lower)	−	Low	Downgraded for moderate/high statistical heterogeneity and imprecision because the total population size was less than 400. No serious risk of bias or indirectness was identified.
Sufentanil consumption	4	105 vs. 110	SMD 0.21 lower (0.48 lower to 0.05 higher)	−	High	Downgraded for imprecision because the total population size was less than 400; upgraded for strong association as reported in the original GRADE assessment. No serious risk of bias, inconsistency, or indirectness was identified.
VAS Resting 0–2 h	10	392 vs. 397	SMD 1.35 lower (1.87–0.83 lower)	−	Low	Downgraded for lack of allocation concealment and lack of blinding, and for moderate/high statistical heterogeneity.
VAS Activity 0–2 h	4	221 vs. 219	SMD 1.50 lower (2.33–0.67 lower)	–	Very low	Downgraded for lack of allocation concealment and lack of blinding, moderate/high statistical heterogeneity, and imprecision because the total population size was less than 400.
Itching	3	14/137 vs. 27/136	RD −0.07 (−0.21 to 0.07)	69 fewer per 1,000 (from 210 fewer to 69 more)	Very low	Downgraded for lack of allocation concealment and lack of blinding, moderate/high statistical heterogeneity, and imprecision because the total population size was less than 400.
Time to first rescue analgesic	2	156 vs. 156	MD 6.10 higher (5.00–7.2 higher)	–	Very low	Downgraded for lack of allocation concealment and lack of blinding, moderate/high statistical heterogeneity, and imprecision because the total population size was less than 400.

### 3.5 Results of meta-analysis

#### 3.5.1 SAPB on PONV

**FIGURE 3:**
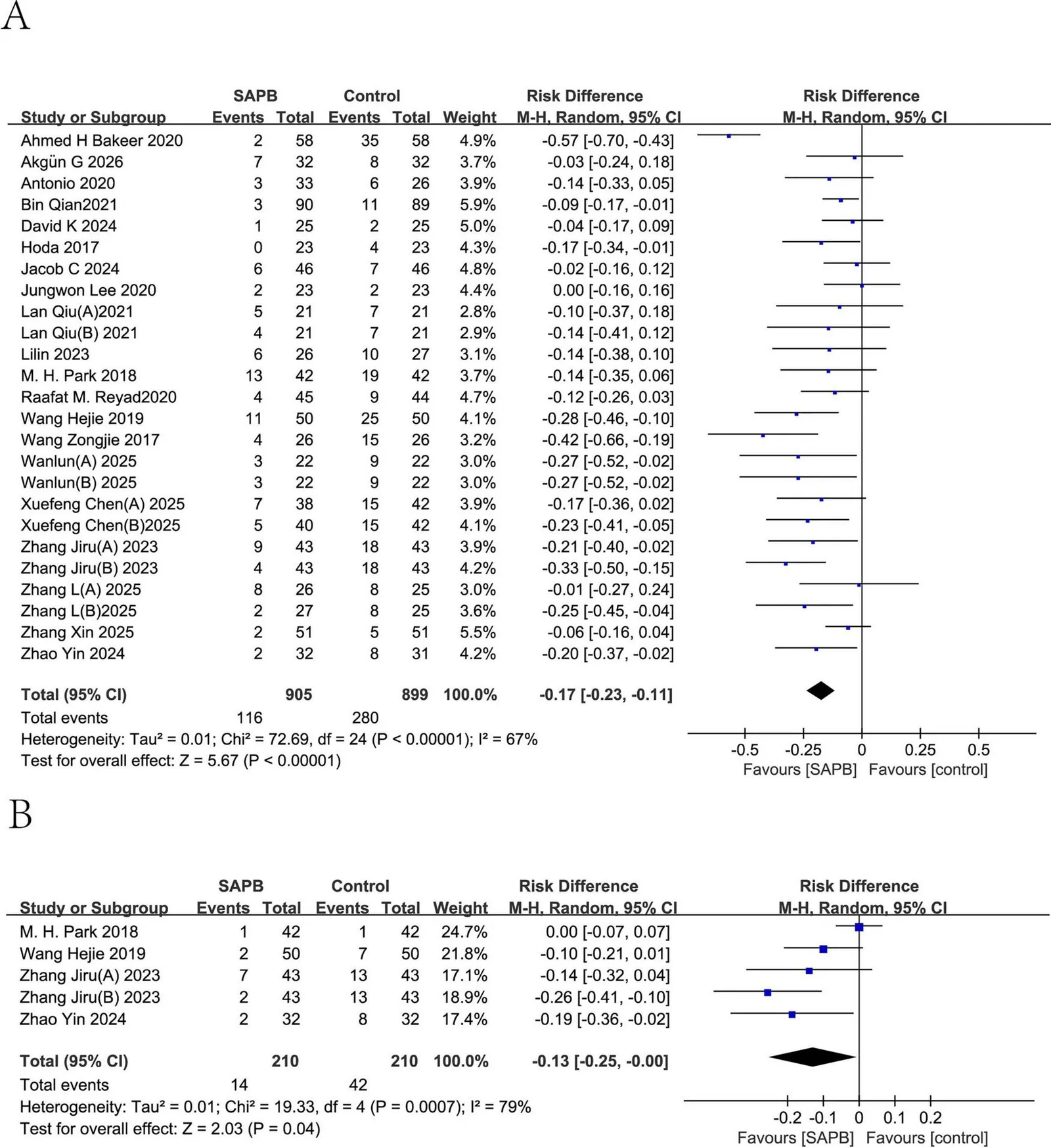
Forest plots showing the effect of serratus anterior plane block (SAPB) on postoperative nausea **(A)** and postoperative vomiting **(B)**. Pooled risk differences (RDs) with 95% confidence intervals (CIs) were calculated using a random-effects model.

**FIGURE 4:**
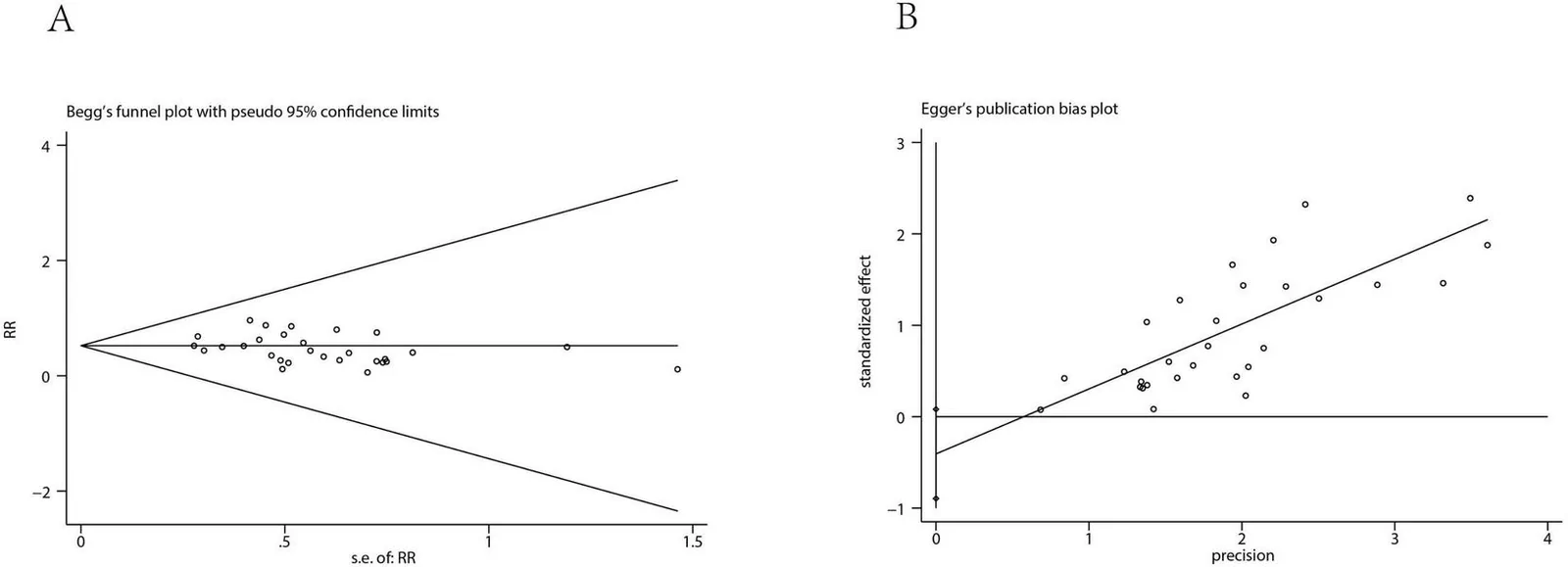
Funnel plot for postoperative nausea. Visual inspection did not show marked asymmetry. Begg’s **(A)** rank correlation test and Egger’s **(B)** regression test did not suggest significant small-study effects.

The effectiveness of SAPB in preventing postoperative nausea and vomiting was evaluated using a random-effects model. For postoperative nausea, 25 analyzable comparisons from 20 trials (12–31) were included, with 905 patients in the SAPB group and 899 in the control group. The incidence of nausea was significantly lower in the SAPB group than in the control group, with a pooled risk difference of −0.17 (95% CI: −0.23 to −0.11; *P* < 0.00001). Substantial heterogeneity was observed for nausea outcomes (*I*^2^ = 67%). For postoperative vomiting, 4 trials involving 420 participants were included, with 210 patients in each group. SAPB may reduce postoperative vomiting, but this finding should be interpreted cautiously because the upper limit of the 95% confidence interval approached the null value (pooled RD = −0.13, 95% CI: −0.25 to −0.00; *P* = 0.04). High heterogeneity was observed for vomiting outcomes (*I*^2^ = 79%) (Figure 3). The effect of SAPB on nausea was supported by moderate-quality evidence as per GRADE. Publication bias was assessed for postoperative nausea because this outcome included more than 10 study comparisons. Visual inspection of the funnel plot did not show marked asymmetry (Figure 4). Begg’s rank correlation test (*P* = 0.122) and Egger’s regression test (*P* = 0.099) did not suggest significant small-study effects. Formal publication bias testing was not performed for postoperative vomiting because fewer than 10 studies reported this outcome, which limited the reliability of funnel plot asymmetry tests. Additionally, factors influencing nausea and vomiting were further explored through subgroup analyses.

#### 3.5.2 Subgroup analysis

##### 3.5.2.1 Type of local anesthetic

**FIGURE 5:**
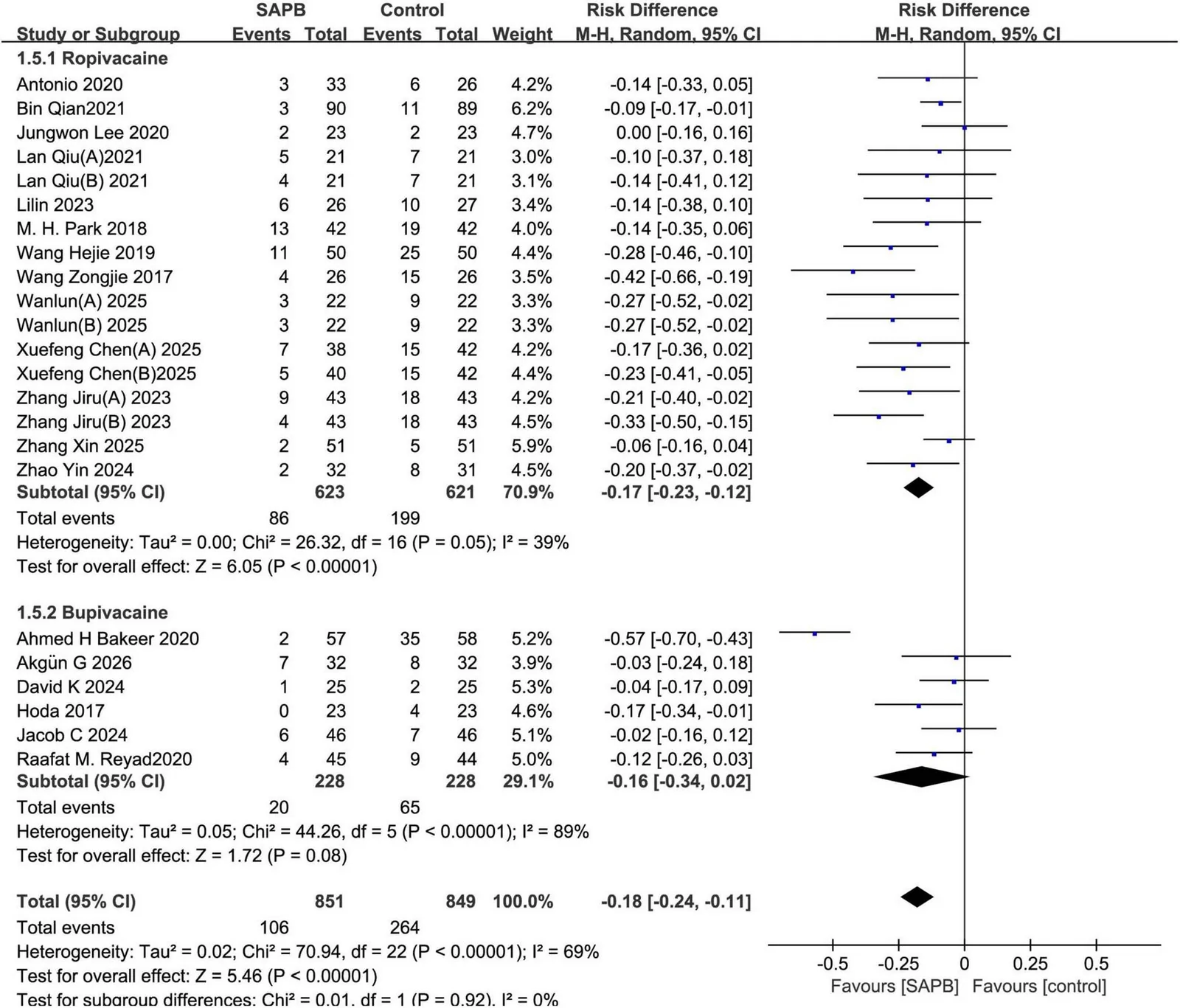
Subgroup analysis of the effect of SAPB on postoperative nausea according to local anesthetic type: ropivacaine versus bupivacaine. Pooled RDs with 95% CIs were calculated using a random-effects model.

Subgroup analysis according to the type of local anesthetic showed that SAPB significantly reduced the incidence of postoperative nausea in the ropivacaine subgroup. Fourteen trials (14, 15, 18–21, 24–31) involving 1,244 participants were included in this subgroup, with 623 patients in the SAPB group and 621 in the control group. The pooled risk difference was −0.17 (95% CI: −0.23 to −0.12; *P* < 0.00001), with moderate heterogeneity (*I*^2^ = 39%). In the bupivacaine subgroup, six trials (12, 13, 16, 17, 22, 23) involving 456 participants were included, with 228 patients in each group. Although the pooled effect also favored SAPB, the reduction in nausea was not statistically significant (RD = −0.16, 95% CI: −0.34–0.02; *P* = 0.08), and substantial heterogeneity was observed (*I*^2^ = 89%). Overall, there was no significant difference between the ropivacaine and bupivacaine subgroups (*P* = 0.92; *I*^2^ = 0%), suggesting that the type of local anesthetic did not significantly modify the effect of SAPB on postoperative nausea (Figure 5).

##### 3.5.2.2 Local anesthetic dosage

**FIGURE 6:**
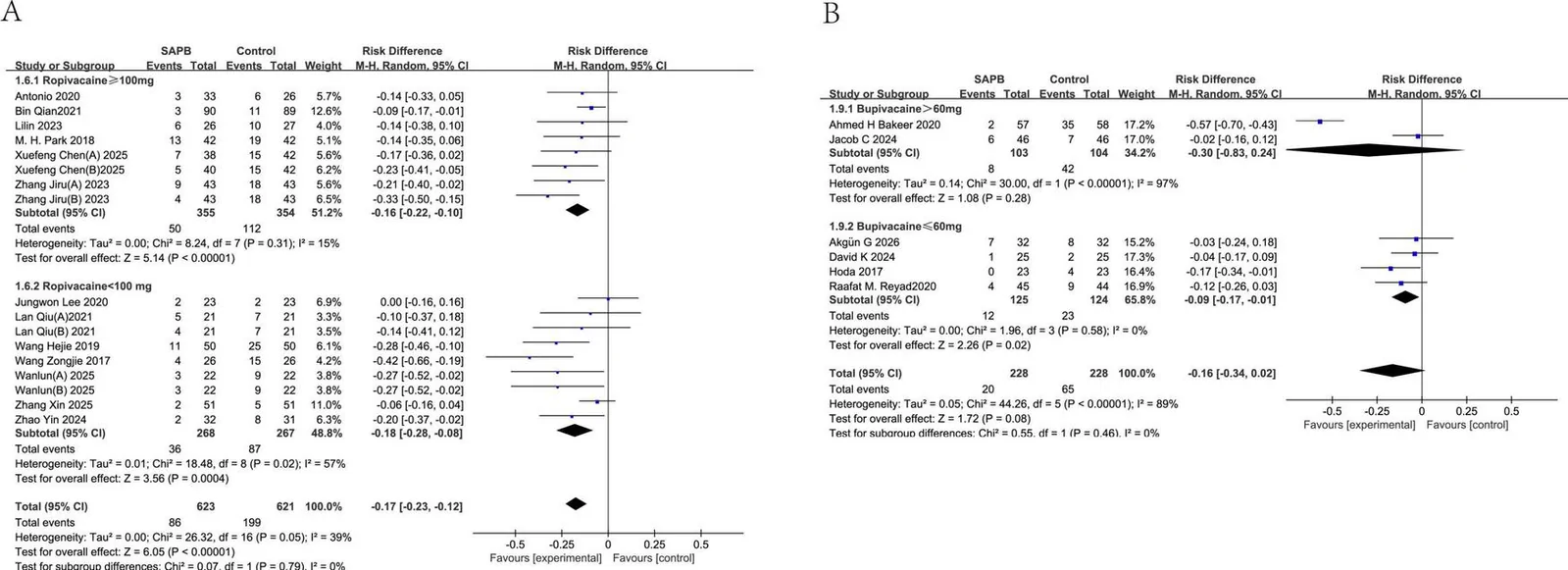
Subgroup analysis of the effect of SAPB on postoperative nausea according to local anesthetic dose. **(A)** Ropivacaine dose ≥ 100 mg versus < 100 mg. **(B)** Bupivacaine dose ≤ 60 mg versus > 60 mg.

Dose-based subgroup analysis showed that SAPB significantly reduced postoperative nausea in both ropivacaine subgroups. In the ≥ 100 mg subgroup, 6 trials (14, 15, 20, 21, 27, 28) with 709 participants showed a pooled RD of −0.16 (95% CI: −0.22 to −0.10; *P* < 0.00001; *I*^2^ = 15%). In the < 100 mg subgroup, 8 trials (18, 19, 24–26, 29–31) with 535 participants also showed a significant reduction (RD = −0.18, 95% CI: −0.28 to −0.08; *P* = 0.0004; *I*^2^ = 57%). No significant difference was found between ropivacaine dose subgroups (*P* = 0.79) (Figure 6A).

For bupivacaine, SAPB significantly reduced nausea in the ≤ 60 mg subgroup, based on 3 trials (13, 16, 23) with 249 participants (RD = −0.09, 95% CI: −0.17 to −0.01; *P* = 0.02; *I*^2^ = 0%). However, the effect was not significant in the > 60 mg subgroup, which included 2 trials (12, 17) with 207 participants (RD = −0.30, 95% CI: −0.83 to 0.24; *P* = 0.28; *I*^2^ = 97%). No significant subgroup difference was observed between bupivacaine dose categories (*P* = 0.46) (Figure 6B).

##### 3.5.2.3 Local anesthetic concentration

**FIGURE 7:**
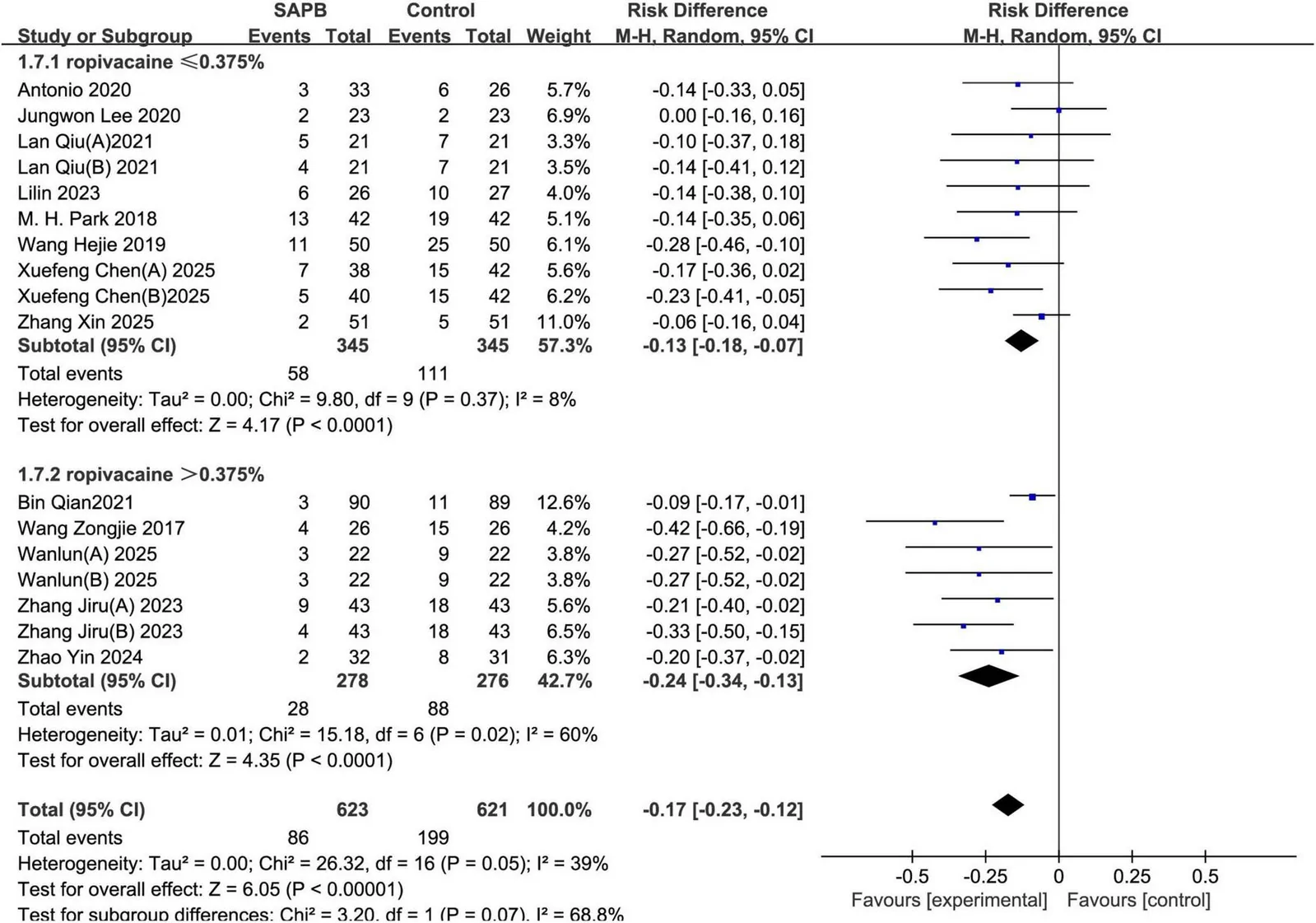
Subgroup analysis of the effect of SAPB on postoperative nausea according to ropivacaine concentration: ≤ 0.375% versus > 0.375%.

Subgroup analysis according to ropivacaine concentration showed that SAPB significantly reduced the incidence of postoperative nausea in both concentration subgroups. In the ropivacaine ≤ 0.375% subgroup, 9 trials (14, 18–21, 24, 27, 29, 30) involving 690 participants were included, with 345 patients in the SAPB group and 345 in the control group. The pooled risk difference was −0.13 (95% CI: −0.18 to −0.07; *P* < 0.0001), with low heterogeneity (*I*^2^ = 8%). In the ropivacaine > 0.375% subgroup, five trials (15, 25, 26, 28, 31) involving 554 participants were included, with 278 patients in the SAPB group and 276 in the control group. SAPB was also associated with a significant reduction in postoperative nausea in this subgroup, with a pooled risk difference of −0.24 (95% CI: −0.34 to −0.13; *P* < 0.0001), although moderate heterogeneity was observed (*I*^2^ = 60%). The test for subgroup differences did not reach statistical significance (*P* = 0.07), suggesting that ropivacaine concentration did not significantly modify the effect of SAPB on postoperative nausea (Figure 7).

##### 3.5.2.4 Operation type

**FIGURE 8:**
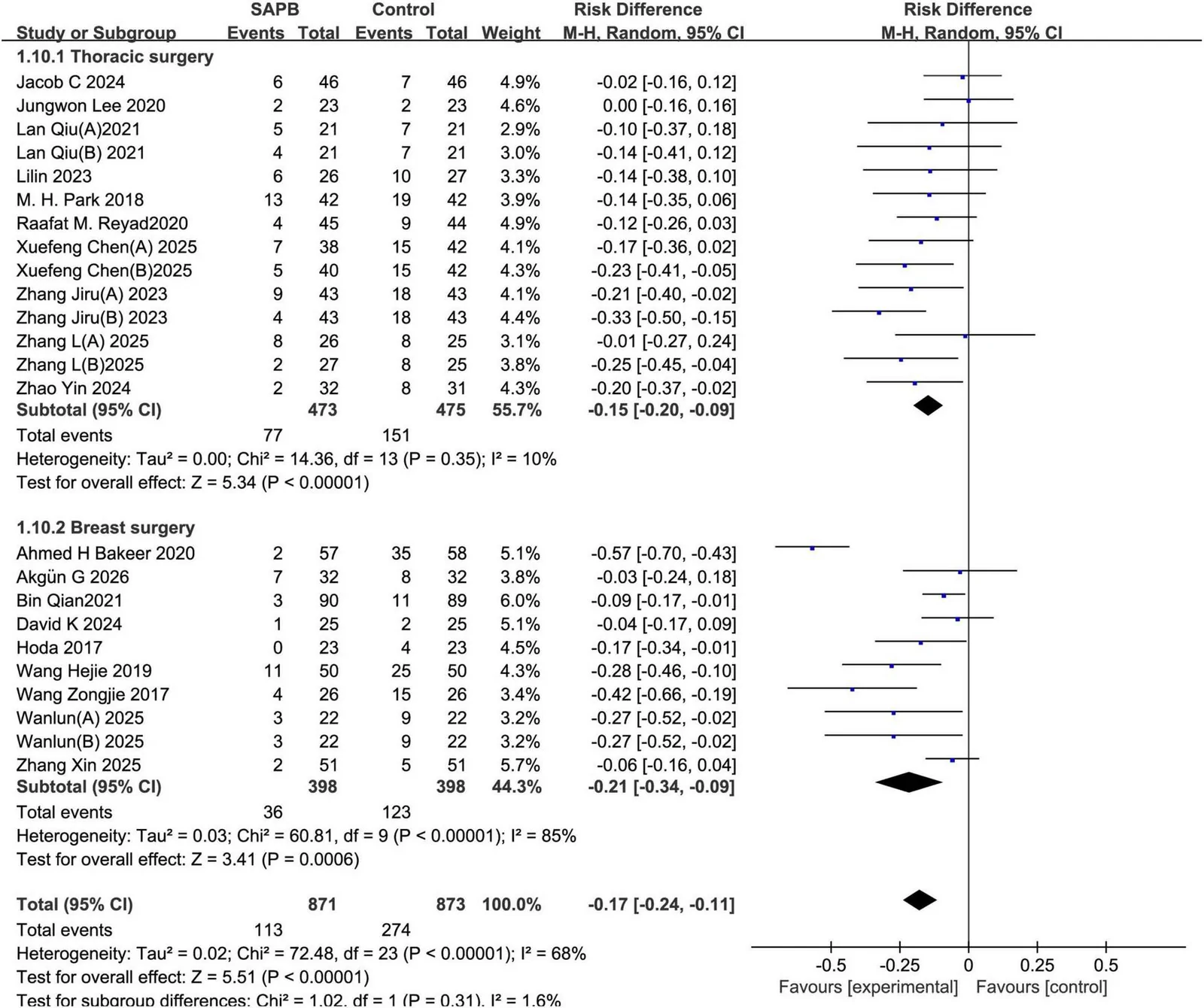
Subgroup analysis of the effect of SAPB on postoperative nausea according to surgical category: breast surgery versus thoracic surgery.

The use of SAPB was associated with a significant reduction in postoperative nausea in both breast and thoracic surgery subgroups. In the thoracic surgery subgroup, 10 trials (17–22, 27–29, 31) involving 948 participants were included, with 473 patients in the SAPB group and 475 in the control group. SAPB significantly reduced the incidence of nausea, with a pooled risk difference of −0.15 (95% CI: −0.20 to −0.09; *P* < 0.00001), and low heterogeneity was observed (*I*^2^ = 10%). In the breast surgery subgroup, 8 trials (12, 13, 15, 16, 23–26, 30) involving 796 participants were included, with 398 patients in each group. SAPB was also associated with a significant reduction in nausea, with a pooled risk difference of −0.21 (95% CI: −0.34 to −0.09; *P* = 0.0006), although substantial heterogeneity was present (*I*^2^ = 85%). The test for subgroup differences was not significant (*P* = 0.31), indicating that the effect of SAPB on postoperative nausea did not differ significantly between thoracic and breast surgery (Figure 8).

##### 3.5.2.5 Timing of SAPB administration

**FIGURE 9:**
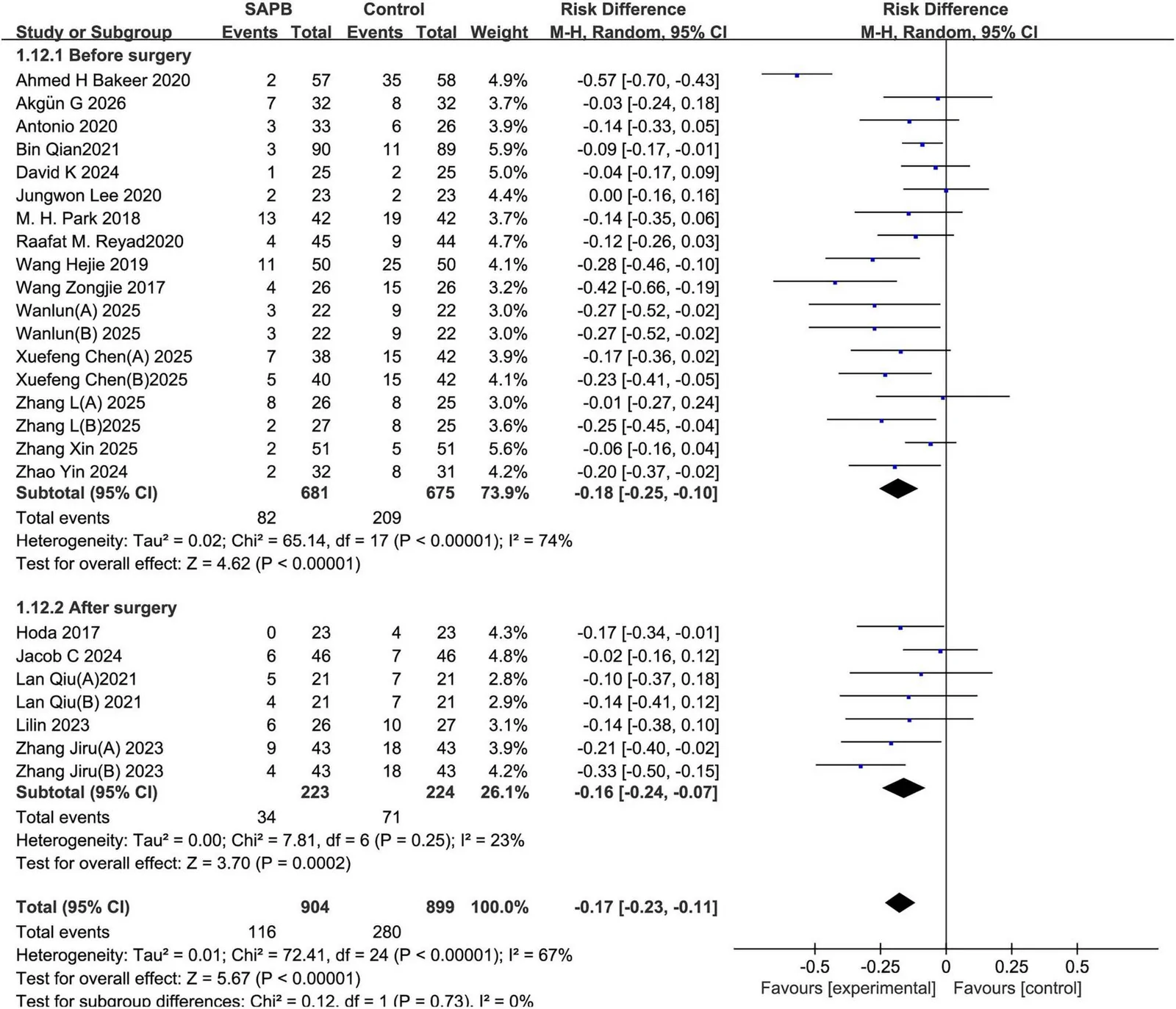
Subgroup analysis of the effect of SAPB on postoperative nausea according to timing of block administration: before surgery versus after surgery.

The reduction in PONV associated with SAPB was consistent regardless of the timing of block administration. When SAPB was performed before surgery, it significantly reduced the incidence of PONV compared with control treatment (pooled RD of 18 trials: −0.18, 95% CI: −0.25 to −0.10). A similar effect was observed when SAPB was administered after surgery (pooled RD of 7 trials: −0.16, 95% CI: −0.24 to −0.07). The test for subgroup differences was not significant (*P* = 0.73), suggesting that the timing of SAPB administration did not significantly influence its preventive effect on PONV (Figure 9).

##### 3.5.2.6 Superficial versus deep SAPB

**FIGURE 10:**
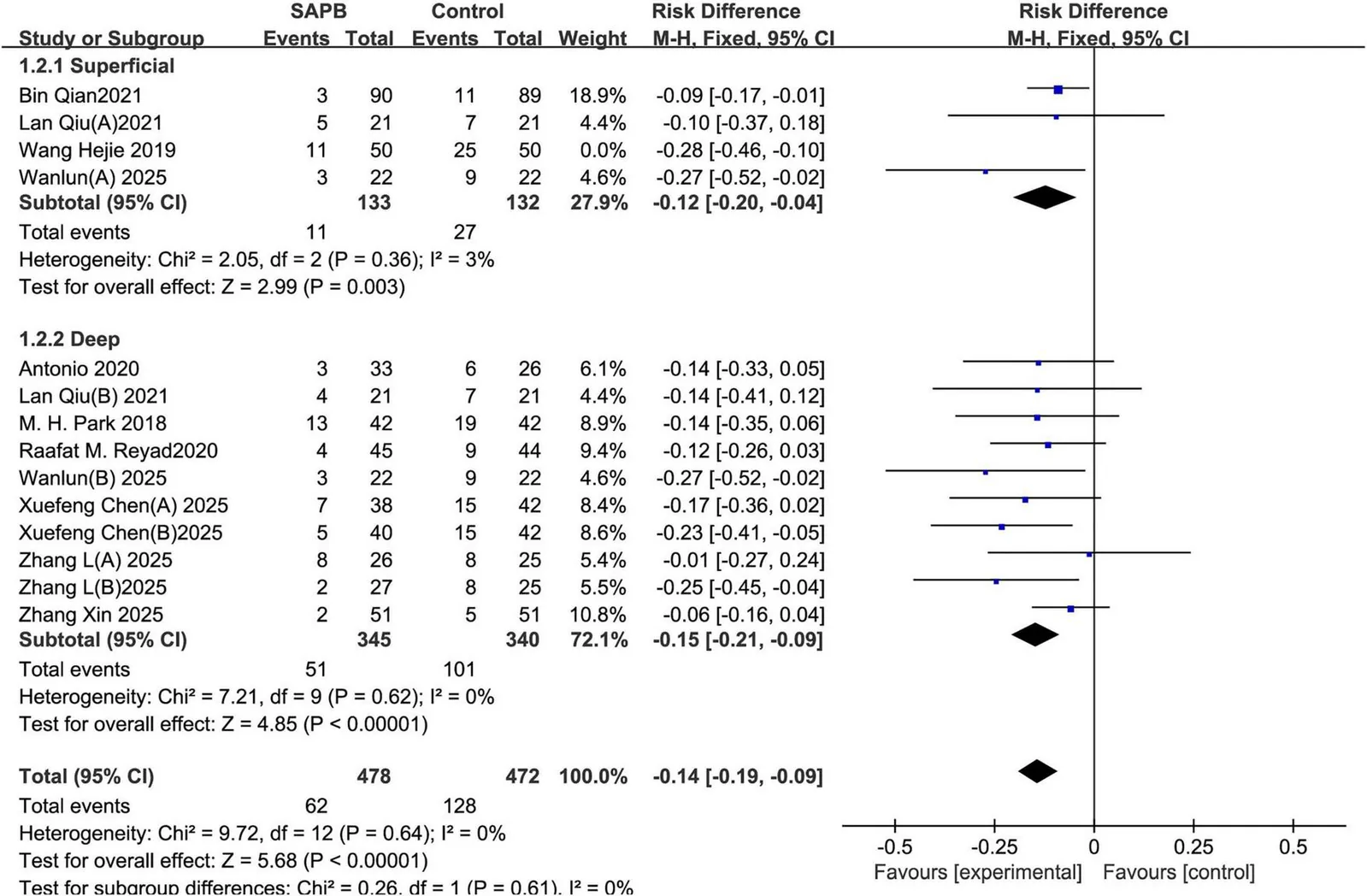
Exploratory subgroup analysis of the effect of SAPB on postoperative nausea according to block plane: superficial SAPB versus deep SAPB.

An exploratory subgroup analysis was performed for studies that clearly reported the SAPB plane as superficial or deep. In the superficial SAPB subgroup, SAPB significantly reduced postoperative nausea compared with control treatment, with a pooled RD of −0.12 (95% CI: −0.20 to −0.04). A similar reduction was observed in the deep SAPB subgroup, with a pooled RD of −0.15 (95% CI: −0.21 to −0.09). The direction of effect favored SAPB in both subgroups, and no significant subgroup difference was detected (*P* = 0.61). However, the number of studies with clearly reported SAPB plane was limited. Therefore, these findings should be interpreted cautiously and should not be used to infer the superiority of one SAPB plane over the other (Figure 10).

#### 3.5.3 Antiemetic efficacy outcomes

All included studies failed to report the administration of rescue antiemetics. However, considering its clinical relevance, future studies should incorporate this outcome into their design. Its absence limits the applicability of the findings to real-world practice.

#### 3.5.4 VAS rest at 0–2, 4–6, 24, and 48 h

VAS scores at rest were significantly reduced with SAPB across all postoperative time points. At 0–2 h postoperatively, 8 trials (14, 15, 19, 20, 22, 24, 25, 28) involving 789 participants were included, with 392 patients in the SAPB group and 397 in the control group. SAPB significantly reduced resting VAS scores, with a pooled SMD of −1.35 (95% CI: −1.87 to −0.83; *P* < 0.00001), although substantial heterogeneity was observed (*I*^2^ = 91%). At 4–6 h, the pooled analysis included 8 trials (15, 19, 20, 22–25, 28) involving 678 participants, with 340 patients in the SAPB group and 338 in the control group; SAPB was also associated with lower resting VAS scores (SMD = −1.31, 95% CI: −1.91 to −0.71; *P* < 0.0001), with substantial heterogeneity (*I*^2^ = 92%). At 24 h, 9 trials (14, 15, 19, 22–25, 28, 30) involving 736 participants were included, and SAPB significantly reduced resting VAS scores (SMD = −1.44, 95% CI: −2.05 to −0.84; *P* < 0.00001; *I*^2^ = 92%). A significant reduction was also observed at 48 h, based on 3 trials (24, 25, 28) involving 324 participants (SMD = −1.59, 95% CI: −2.61 to −0.56; *P* = 0.002; *I*^2^ = 94%) (Supplementary Figure 1).

#### 3.5.5 VAS activity at 0–2, 4–6, 24 h

SAPB was also associated with reduced VAS scores during activity. Significant reductions were observed at 0–2 h (pooled SMD from 3 trials (15, 22, 28): −1.50, 95% CI: −2.33 to −0.67), 4–6 h (pooled SMD from 4 trials (13, 15, 22, 28): −1.33, 95% CI: −2.07 to −0.60), and 24 h (pooled SMD from 4 trials (15, 22, 28, 30): −1.11, 95% CI: −1.69 to −0.52) (Supplementary Figure 2).

#### 3.5.6 Consumption of fentanyl, sufentanil, and remifentanil

SAPB was associated with reduced postoperative opioid consumption. A significant reduction was observed for fentanyl consumption, based on 4 trials (12, 13, 21, 22) (pooled SMD = −1.08, 95% CI: −1.86 to −0.31) (Supplementary Figure 3A). SAPB also significantly reduced remifentanil consumption, based on 5 trials (19, 24, 28, 29, 31) (pooled SMD = −2.14, 95% CI: −3.25 to −1.03) (Supplementary Figure 3C). In contrast, although sufentanil consumption tended to be lower in the SAPB group, the difference was not statistically significant, based on 3 trials (14, 20, 21) (pooled SMD = −0.21, 95% CI: −0.48 to 0.05) (Supplementary Figure 3B).

#### 3.5.7 Length of hospital stay, time to first rescue analgesia, and the incidence of headache and itching

Application of SAPB was associated with a shorter length of hospital stay. Based on 4 trials (22, 24, 28, 29), SAPB reduced hospital stay by 0.71 days compared with control treatment (MD = −0.71 days, 95% CI: −1.07 to −0.34; *P* = 0.0001; *I*^2^ = 54%) (Supplementary Figure 4A), corresponding to an absolute reduction of approximately 17 h. Because no universally accepted minimal clinically important difference exists for hospital stay in this mixed surgical population, this finding was interpreted descriptively. SAPB also prolonged the time to first rescue analgesia, based on 2 trials (12, 26) involving 156 patients in each group (pooled MD = 6.10, 95% CI: 5.00–7.20; *P* < 0.00001; *I*^2^ = 98%) (Supplementary Figure 4B). In addition, SAPB reduced the incidence of headache, based on 5 trials (15, 21, 22, 29, 31) involving 255 SAPB patients and 256 control patients (pooled RD = −0.09, 95% CI: −0.18 to −0.00; *P* = 0.04; *I*^2^ = 78%) (Supplementary Figure 4C). However, no significant difference was observed in the incidence of itching, based on 3 trials (21, 22, 24) involving 137 SAPB patients and 136 control patients (pooled RD = −0.07, 95% CI: −0.21 to 0.07; *P* = 0.34; *I*^2^ = 84%) (Supplementary Figure 4D).

#### 3.5.8 Trial sequential analysis

**FIGURE 11:**
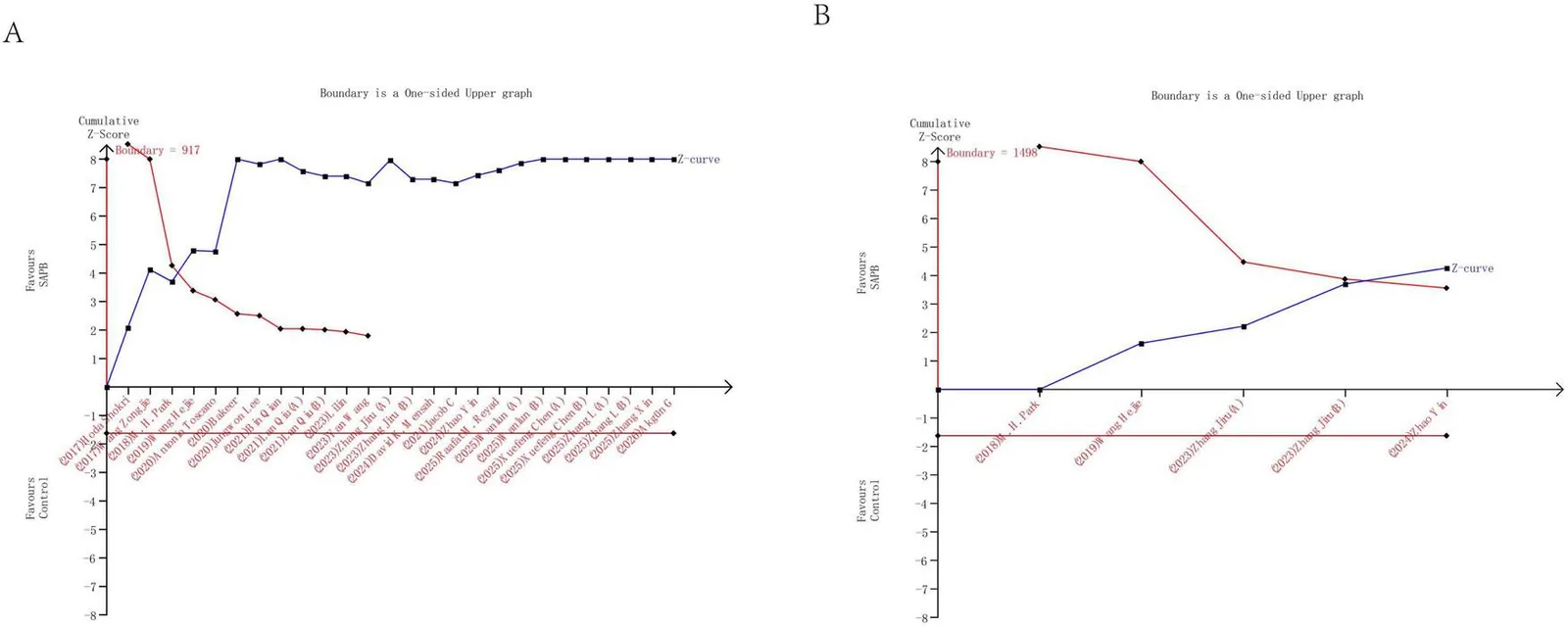
Trial sequential analysis of cumulative evidence for the effect of SAPB on postoperative nausea **(A)** and postoperative vomiting **(B)**. TSA supported the robustness of the pooled finding for postoperative nausea, whereas the vomiting outcome should be interpreted cautiously because of the borderline pooled confidence interval and limited number of events.

Using data from all 20 randomized controlled trials (1,651 participants), TSA was carried out for nausea and vomiting outcomes. With a predefined 5% type I error and 20% type II error, the diversity-adjusted required information size was calculated. For postoperative nausea, TSA supported the robustness of the pooled finding. For postoperative vomiting, the result should be interpreted more cautiously because the pooled confidence interval was borderline and the number of vomiting events was limited (Figure 11).

#### 3.5.9 Sensitivity analysis

To evaluate the robustness of the pooled risk difference for postoperative nausea, a leave-one-out sensitivity analysis was performed. Each study was sequentially omitted from the meta-analysis, and the pooled RD was recalculated using a random-effects inverse-variance model. As shown in Supplementary Figure 5, omission of any single study did not materially change the overall effect estimate. The recalculated pooled RDs ranged from −0.18 to −0.14, and all corresponding 95% CIs remained below the null value of 0. This indicates that the protective effect of SAPB on postoperative nausea was not driven by any individual study. Overall, the sensitivity analysis supports the robustness and stability of the pooled result. In addition, a sensitivity analysis excluding studies judged to be at high risk of bias was performed for the primary outcome of postoperative nausea. After exclusion of high-risk studies, the pooled effect remained directionally consistent with the main analysis and continued to favor SAPB (RD = −0.18, 95% CI: −0.25 to −0.10; *P* < 0.00001). The heterogeneity remained substantial (*I*^2^ = 73%), suggesting that the overall finding was not solely driven by studies at high risk of bias but that residual between-study heterogeneity persisted. Therefore, this sensitivity analysis supports the robustness of the direction of effect, while the magnitude of the effect should still be interpreted cautiously (Supplementary Figure 6).

## 4 Discussion

The main findings of this meta-analysis suggest that SAPB was associated with a lower incidence of postoperative nausea and may reduce postoperative vomiting, together with improved postoperative analgesia and reduced opioid consumption. This benefit was generally observed across breast and thoracic surgery populations, supporting the clinical relevance of SAPB in chest wall–related procedures. Subgroup analyses indicated that the reduction in nausea was broadly consistent across ropivacaine dose and concentration categories, while bupivacaine showed a significant effect mainly at doses ≤ 60 mg. In addition, SAPB reduced pain scores at rest and during movement at several postoperative time points, decreased fentanyl and remifentanil consumption, shortened hospital stay, delayed the need for rescue analgesia, and reduced headache incidence. The reduction in hospital stay was approximately 0.71 days, corresponding to about 17 h; because no universally accepted MCID has been established for hospital stay in this mixed surgical population, this result was interpreted descriptively. However, the effects on sufentanil consumption, pruritus, and hypotension were not statistically conclusive. From a clinical perspective, the observed reductions in opioid consumption and early postoperative pain scores may be meaningful, as they approach or exceed commonly used thresholds for clinically important improvement, including an approximately 30% reduction in opioid consumption and an approximately 1-point reduction in visual analog scale pain scores. However, because these thresholds were used for contextual interpretation rather than as predefined primary decision criteria, the clinical implications should be interpreted cautiously and in conjunction with the certainty of evidence and between-study heterogeneity. While our analysis confirms a postoperative VAS score reduction at 48 h attributed to SAPB, the long-term analgesic potential of this technique remains to be established.

Comparison with previous SAPB studies showed that our findings were generally consistent with the analgesic and opioid-sparing effects reported in earlier randomized trials. In breast surgery, several studies reported lower postoperative nausea rates after SAPB than after saline or no-block control, including those by Bakeer et al. (13), Wang et al. (24), Wang et al. (25), and Zhang et al. (30). Similar trends were also observed in thoracic surgery trials, including those by Reyad et al. (22), Chen et al. (27), Zhang et al. (28), and Zhao et al. (31). These findings support the hypothesis that SAPB may reduce PONV mainly through improved regional analgesia and reduced perioperative opioid exposure. However, not all individual studies showed a clear benefit for PONV. For example, Akgün et al. (13) and Jackson et al. (17) reported only small between-group differences in postoperative nausea, while Lee et al. (18) reported identical nausea rates between the SAPB and control groups. Vomiting outcomes were also less consistent, probably because of the low number of events. These neutral or inconsistent findings may be explained by small sample sizes, differences in baseline PONV risk, variability in prophylactic antiemetic regimens, differences in intraoperative and postoperative opioid protocols, heterogeneous surgical populations, different timing of SAPB administration, and inconsistent definitions or observation windows for nausea and vomiting. Therefore, although the overall pooled estimate favored SAPB, the findings should be interpreted as evidence of a probable opioid-sparing and analgesia-mediated reduction in PONV rather than as proof of a direct antiemetic effect of SAPB.

PONV frequently occurs after anesthesia, representing an important concern in the perioperative period (32). PONV arises due to three distinct risk elements: patient-specific factors, the chosen anesthesia technique, and the details of the surgical procedure (33). The administration of opioids during and after surgery introduces risks related to anesthesia. PONV remains a major issue, with the type of anesthetic used potentially influencing its occurrence, despite extensive research (34). In this review, we examine adjunctive methods for controlling PONV, with special attention to the efficacy of SAPB assessed through this meta-analysis.

The use of opioids can effectively control surgical pain, but it is associated with potential side effects, including dizziness, postoperative nausea and vomiting, and respiratory depression (35, 36). Compared with single general anesthesia, combined regional block provides better postoperative analgesia and significantly reduces adverse reactions associated with opioids (37). Therefore, SAPB may contribute to a lower risk of postoperative nausea and vomiting primarily by reducing pain intensity and opioid exposure, although the magnitude of this benefit may vary across surgical settings and perioperative protocols.

The reduction in PONV observed in the present meta-analysis should be interpreted primarily in the context of improved analgesia and opioid sparing rather than as evidence of a direct antiemetic effect of SAPB. SAPB provides regional analgesia of the anterolateral chest wall by blocking the lateral cutaneous branches of the intercostal nerves and related thoracic wall innervation (6). Improved regional analgesia can reduce intraoperative and postoperative opioid requirements, and opioid exposure is a well-established risk factor for PONV (38). Therefore, the most plausible causal pathway is that SAPB reduces postoperative pain, thereby decreasing opioid consumption, which subsequently contributes to a lower incidence of postoperative nausea and possibly vomiting. Other mechanisms may also contribute, but these remain speculative. By attenuating nociceptive input from the surgical field, SAPB may reduce surgical stress responses and anesthetic requirements, both of which may influence postoperative recovery and PONV risk (34, 37, 39, 40). However, the included randomized trials were not designed to isolate a direct antiemetic effect of SAPB, and most did not consistently report rescue antiemetic use, volatile anesthetic exposure, or standardized opioid protocols. Therefore, the observed reduction in PONV should be regarded as likely mediated by analgesic and opioid-sparing effects rather than as proof that SAPB independently prevents nausea or vomiting.

There are many strategies to manage pain after surgery. The most direct options include intravenous injection of opioid analgesics. Pain control of opioids usually requires relatively high doses, which may cause adverse reactions, including respiratory depression, nausea, vomiting and itching (35). Regional analgesia techniques of the chest wall include epidural analgesia (TEA), paravertebral block (PVB), intercostal nerve block and interpleural local anesthesia infusion (41). Among them, TEA and PVB are considered to be the most effective methods for postoperative pain control (42). However, both methods may bring the risk of complications such as pneumothorax, spinal cord injury, hypotension associated with nerve axis block, and respiratory muscle injury (43). In contrast, intercostal catheter analgesia and interpleural drug infusion usually do not provide sufficient pain relief, especially during video-assisted thoracoscopic surgery (VATS). Compared with traditional technologies such as TEA and PVB, SAPB provides a favorable balance between analgesic efficacy and safety, making it particularly suitable for a series of chest wall-related surgeries.

In thoracotomy, the main source of pain comes from the serrated muscle-rib-intercostal nerve complex. This anatomical area is the direct target of SAPB (44), which may weaken the peripheral and central sensitization mechanisms associated with post-thoracotomy pain syndrome (PTPS). SAPB was associated with an opioid-sparing effect, which may partly explain the observed reduction in postoperative nausea and vomiting. Improved pain control also contributes to enhanced postoperative respiratory function, which may further reduce the occurrence of PONV. On the other hand, we also found that SAPB is superior to patient-controlled intravenous analgesia (PCIA) in prolonging the duration of postoperative analgesia and reducing the requirement for rescue analgesics in patients undergoing radical mastectomy for breast cancer. SAPB may serve as a viable alternative to TEA, particularly in patients with coagulation abnormalities, in whom TEA is contraindicated and carries a higher risk of adverse effects. In summary, SAPB was associated with a lower incidence of postoperative nausea in both breast and thoracic surgery subgroups, while the evidence for vomiting was less certain because of the low number of events. This finding is consistent with its anatomical coverage, which primarily targets the innervation of the anterolateral chest wall. Therefore, the application of SAPB in chest wall–related procedures shows strong biological plausibility.

Our analysis indicated that increasing the concentration of ropivacaine beyond 0.5% was associated with a diminished antiemetic benefit of SAPB, whereas bupivacaine at concentrations above 0.5% was linked to a higher likelihood of toxicity. Interestingly, elevated concentrations of bupivacaine were still associated with a reduction in PONV, which may be explained by improved analgesic effectiveness. Nevertheless, PONV outcomes were inconsistently reported across the included studies, which limited the ability to clearly distinguish the relative effects of different anesthetic regimens on pain control versus nausea prevention. Although the need for rescue antiemetic therapy represents a clinically meaningful endpoint, it was not consistently documented in the included trials and therefore could not be incorporated into the pooled analysis. Future studies should prioritize standardized reporting of this outcome to enable more reliable and comprehensive evidence synthesis.

SAPB and PVB represent effective approaches for postoperative analgesia after radical mastectomy. Although PVB has proven efficacy against placebo in thoracic surgeries, concerns over potential complications—such as spinal injury, intraspinal hematoma, and sympathetic blockade effects—may limit its use (42). SAPB, particularly when performed under ultrasound guidance, is a relatively novel technique of regional analgesia for thoracic pain. SAPB was non-inferior to paravertebral block with respect to the incidence of nausea and vomiting, the pain score and opioid consumption. Its growing clinical adoption can be attributed to a more favorable safety profile and the relative simplicity of the technique. Nevertheless, further research is needed to more clearly define its comparative effectiveness and safety against other chest wall blocks. Considering pain following thoracic surgery, including thoracotomy and thoracoscopic procedures, is multifactorial in origin and typically necessitates a multimodal analgesic strategy, so the SAPB gains its popularity due to its low complication rate. As pain is relieved, postoperative respiratory function can be improved, indicating that multimodal analgesia with SAPB plays a positive role in the recovery of oxygenation function and the reduction of pulmonary complications.

Current guidelines highlight several major risk factors for PONV, including female gender, nonsmoking status, postoperative opioids and history of motion sickness. The incidence of PONV is approximately 30% during the perioperative period, making PONV a critical factor affecting postoperative recovery quality and hospital discharge efficiency. Both volatile anesthetics and postoperative opioid use are independent predictors of PONV (34). Moreover, PONV significantly impacts patients, disrupting fluid and electrolyte equilibrium and, in severe cases, leading to aspiration pneumonitis, delayed discharge, increased hospital expenses, and added financial stress (32). Our findings demonstrate that SAPB provides dual benefits: it reduces opioid consumption within the first 24 h (consistent with the duration of local anesthetics) and lowers the incidence of PONV, likely through the opioid-sparing mechanism (10). In addition, SAPB contributes to lower postoperative pain scores, further contributing to PONV reduction. Therefore, SAPB is well aligned with the enhanced recovery after surgery (ERAS) principles due to its favorable safety profile and minimal adverse effects, including multimodal analgesia, opioid reduction, and accelerated recovery. Meanwhile, SAPB exhibits non-inferior efficacy in preventing PONV and may offer potential clinical benefits, especially in patients at high risk of PONV, including female patients, those undergoing breast or thoracic surgery, and individuals requiring opioid administration. These findings are consistent with the established analgesic effect of SAPB and also emphasize its potential role in reducing PONV. In clinical practice, SAPB can be an integral part of a multi-model pain management strategy, which may help reduce the risk of PONV by relieving pain and reducing opioid exposure.

The obvious heterogeneity (*I*^2^ = 73%) observed in nausea results may be attributed to differences in SAPB techniques—such as the type and concentration of local anesthetics—and changes in the surgical environment.. For example, subgroup analyses suggested a greater reduction in PONV with ropivacaine at concentrations ≤ 0.5% and bupivacaine doses > 60 mg at 0.5%. However, these findings should be interpreted cautiously, as they are exploratory and based on a limited number of studies in certain subgroups. Although heterogeneity limits the ability to draw definitive conclusions, it highlights the importance of standardized protocols in future research.

We further explored clinical heterogeneity through subgroup analyses according to surgical category and SAPB plane when clearly reported. The reduction in postoperative nausea was directionally consistent across breast and thoracic surgery, and the exploratory analysis of superficial versus deep SAPB did not provide robust evidence that one plane was superior to the other. However, subgroup analyses according to open versus minimally invasive procedures and single-shot versus continuous SAPB could not be reliably performed because these variables were not consistently or explicitly reported in the included trials. In particular, surgical approach was closely correlated with surgical category, and assigning studies to open or minimally invasive subgroups by inference alone could introduce misclassification bias. Similarly, SAPB administration mode was insufficiently reported in several trials, and the number of continuous SAPB studies was limited. These reporting limitations may partly explain the residual heterogeneity and should be addressed in future randomized trials.

### 4.1 Limitations and suggestions for practice

This study has notable limitations, the foremost of which is the substantial heterogeneity present among the included studies. Statistical analysis revealed significant heterogeneity in the pooled results regarding the association between SAPB and PONV incidence, which may be attributed to multiple confounding factors. Second, the non-uniform antiemetic and opioid regimens lacked standardization in terms of drug types and dosages. More optimized antiemetic protocols (such as ondansetron or combination therapy) and more precise opioid administration may contribute to a reduced incidence of PONV. Third, the study population was characterized by a relatively favorable baseline health status, with limited inclusion of emergency and critically ill patients, which may compromise the external validity and broader generalizability of our results. Furthermore, although subgroup analyses stratified by surgical type, local anesthetic agent, and dosage yielded clinically valuable insights, their interpretation requires caution due to potential methodological limitations. Specifically, some subgroups (e.g., the bupivacaine < 100 mg subgroup) included only a small number of studies, which may increase the risk of Type II error and reduce the statistical power and generalizability of the corresponding results. We restricted the primary analysis to trials comparing SAPB with placebo, sham saline injection, no regional block, or no intervention, in order to estimate the effect of SAPB relative to an inactive or usual-care control. Trials comparing SAPB only with another active regional anesthetic technique were excluded from the primary analysis because such comparisons address a different clinical question and involve heterogeneous comparator techniques. Therefore, the present findings should not be interpreted as evidence that SAPB is superior or inferior to other regional anesthetic techniques. Future direct-comparison meta-analyses or network meta-analyses are needed to compare SAPB with other regional blocks, such as paravertebral block, erector spinae plane block, pectoral nerve block, and intercostal nerve block. Further network meta-analyses are warranted to compare and rank the efficacy of SAPB against other available regional block strategies. Finally, according to the GRADE assessment, the certainty of evidence varied across outcomes, ranging from moderate to very low. The main reasons for downgrading were risk of bias, statistical heterogeneity, and imprecision.

## 5 Conclusion and recommendations

In summary, SAPB appears to reduce postoperative opioid requirements and improve analgesia in patients undergoing breast and thoracic surgery. These analgesic and opioid-sparing effects may be associated with a reduced incidence of postoperative nausea and may contribute to enhanced recovery. However, the present evidence does not establish an independent direct antiemetic effect of SAPB. Further high-quality randomized trials with standardized opioid and antiemetic protocols are needed to clarify the causal pathway between SAPB, opioid reduction, and PONV.

## Data Availability

The original contributions presented in this study are included in the article/Supplementary material, further inquiries can be directed to the corresponding author.

## Supplementary material

Supplementary Figure 1 Forest plots of visual analog scale (VAS) pain scores at rest at 0–2 h **(A)**, 4–6 h **(B)**, 24 h **(C)**, and 48 h **(D)** postoperatively. Pooled standardized mean differences (SMDs) with 95% CIs were calculated using a random-effects model.

Supplementary Figure 2 Forest plots of VAS pain scores during movement at 0–2 h **(A)**, 4–6 h **(B)**, and 24 h **(C)** postoperatively. Pooled SMDs with 95% CIs were calculated using a random-effects model.

Supplementary Figure 3 Forest plots of perioperative opioid consumption. **(A)** Fentanyl consumption. **(B)** Sufentanil consumption. **(C)** Remifentanil consumption. Pooled SMDs with 95% CIs were calculated using a random-effects model.

Supplementary Figure 4 Forest plots of secondary outcomes. **(A)** Length of hospital stay, reported as mean difference (MD) in days. **(B)** Time to first rescue analgesia, reported as MD in hours. **(C)** Incidence of headache, reported as RD. **(D)** Incidence of itching, reported as RD.

Supplementary Figure 5 Leave-one-out sensitivity analysis for postoperative nausea. Each study was sequentially omitted, and the pooled RD was recalculated to assess the robustness of the overall effect estimate.

Supplementary Figure 6 Sensitivity analysis excluding studies judged to be at high risk of bias for postoperative nausea.
