# Supplementary material for: Efficacy of serratus anterior plane block on postoperative nausea and vomiting: a meta-analysis of randomized controlled trial
Source: Front Med (Lausanne). 2026 Aug 12;13:1867333. doi: 10.3389/fmed.2026.1867333 (PMC13506783; doi:10.3389/fmed.2026.1867333)
Supplement: Supplementary file 1 [file Table_1.docx]

**Supplementary Table 1. Detailed search strategies for all databases**

| **Database** | **Search Date** | **Language** | **Search Strategy** |
| --- | --- | --- | --- |
| PubMed | Up to April 20, 2026 | No restriction | ("Serratus anterior plane block"[tiab] OR "Serratus anterior anesthesia"[tiab] OR "Serratus anterior analgesia"[tiab] OR SAPB[tiab] OR "Serratus anterior plane block"[Mesh]) AND ("postoperative nausea and vomiting"[tiab] OR PONV[tiab] OR "nausea"[tiab] OR "vomiting"[tiab] OR "postoperative nausea and vomiting"[Mesh]) AND ("randomized controlled trial"[tiab] OR RCT[tiab] OR "randomly assigned"[tiab]) |
| Embase | Up to April 20, 2026 | No restriction | ('serratus anterior plane block'/exp OR 'serratus anterior plane block':ti,ab OR 'serratus anterior anesthesia':ti,ab OR 'serratus anterior analgesia':ti,ab OR 'SAPB':ti,ab) AND ('postoperative nausea and vomiting':ti,ab OR 'PONV':ti,ab OR 'nausea':ti,ab OR 'vomiting':ti,ab) AND ('randomized controlled trial':ti,ab OR 'RCT':ti,ab OR 'randomly assigned':ti,ab) |
| Cochrane Library (CENTRAL) | Up to April 20, 2026 | No restriction | ("Serratus anterior plane block" OR "Serratus anterior anesthesia" OR "Serratus anterior analgesia" OR SAPB) AND ("postoperative nausea and vomiting" OR PONV OR "nausea" OR "vomiting") AND ("randomized controlled trial" OR RCT OR "randomly assigned") |
| CNKI | Up to April 20, 2026 | No restriction | (“前锯肌平面阻滞” OR “前锯肌阻滞” OR “锯肌平面阻滞” OR “SAPB”) AND (“术后恶心呕吐” OR “恶心” OR “呕吐” OR “PONV”) AND (“随机对照试验” OR “随机” OR “RCT”) |
