# Supplementary material for: Efficacy of serratus anterior plane block on postoperative nausea and vomiting: a meta-analysis of randomized controlled trial
Source: Front Med (Lausanne). 2026 Aug 12;13:1867333. doi: 10.3389/fmed.2026.1867333 (PMC13506783; doi:10.3389/fmed.2026.1867333)
Supplement: Supplementary file 2 [file Table_2.docx]

Supplementary Table 2. Domain-level risk-of-bias assessment using the Cochrane Risk of Bias tool, version 1 (RoB 1)

| Study | Random sequence generation | Allocation concealment | Blinding of participants and personnel | Incomplete outcome data | Selective reporting | Other bias | Overall judgment |
| --- | --- | --- | --- | --- | --- | --- | --- |
| Ahmed H Bakeer 2020 | Low risk | Low risk | Low risk | Low risk | Low risk | Low risk | Low risk |
| Akgün G 2026 | Low risk | Low risk | Low risk | Low risk | Low risk | Low risk | Low risk |
| Antonio 2020 | Unclear risk | Unclear risk | High risk | Low risk | Low risk | Low risk | High risk |
| Bin Qian 2021 | Low risk | Low risk | Low risk | Low risk | Low risk | Low risk | Low risk |
| David K 2024 | Low risk | Low risk | Low risk | Low risk | Low risk | Low risk | Low risk |
| Hoda 2017 | Low risk | Unclear risk | Unclear risk | Low risk | Low risk | Low risk | Unclear risk |
| Jacob C 2024 | Low risk | Low risk | Low risk | Low risk | Low risk | Low risk | Low risk |
| Jungwon Lee 2020 | Low risk | Unclear risk | Low risk | Low risk | Low risk | Low risk | Unclear risk |
| Lan Qiu 2021 | Low risk | Unclear risk | Unclear risk | Low risk | Low risk | Low risk | Unclear risk |
| Lilin 2023 | Low risk | Unclear risk | High risk | Low risk | Low risk | Low risk | High risk |
| M. H. Park 2018 | Low risk | Low risk | Low risk | Low risk | Low risk | Low risk | Low risk |
| Raafat M. Reyad 2020 | Low risk | Low risk | Unclear risk | Low risk | Low risk | Low risk | Unclear risk |
| Wang Hejie 2019 | Low risk | Low risk | Unclear risk | Low risk | Low risk | Low risk | Unclear risk |
| Wang Zongjie 2017 | Low risk | Low risk | Unclear risk | Low risk | Low risk | Low risk | Unclear risk |
| Wanlun 2025 | Low risk | Low risk | High risk | Low risk | Low risk | Low risk | High risk |
| Xuefeng Chen 2025 | Low risk | Low risk | Unclear risk | Low risk | Low risk | Low risk | Unclear risk |
| Zhang Jiru 2023 | Low risk | Low risk | Unclear risk | Low risk | Low risk | Low risk | Unclear risk |
| Zhang L 2025 | Low risk | Low risk | Low risk | Low risk | Low risk | Low risk | Low risk |
| Zhang Xin 2025 | Low risk | Unclear risk | High risk | Low risk | Low risk | Low risk | High risk |
| Zhao Yin 2024 | Low risk | Unclear risk | High risk | Low risk | Low risk | Low risk | High risk |
